# Endostatin Stimulates Proliferation and Migration of Myofibroblasts Isolated from Myocardial Infarction Model Rats

**DOI:** 10.3390/ijms19030741

**Published:** 2018-03-06

**Authors:** Akira Sugiyama, Yuka Hirano, Muneyoshi Okada, Hideyuki Yamawaki

**Affiliations:** Laboratory of Veterinary Pharmacology, School of Veterinary Medicine, Kitasato University, Higashi 23 bancho 35-1, Towada City, Aomori 034-8628, Japan; dv17003@st.kitasato-u.ac.jp (A.S.); vm11502o@st.kitasato-u.ac.jp (Y.H.); yamawaki@vmas.kitasato-u.ac.jp (H.Y.)

**Keywords:** endostatin, myofibroblast, myocardial infarction

## Abstract

Myofibroblasts contribute to the healing of infarcted areas after myocardial infarction through proliferation, migration, and production of extracellular matrix (ECM). Expression of endostatin, a cleaved fragment of type XVIII collagen, increases in the heart tissue of an experimental myocardial infarction model. In the present study, we examined the effect of endostatin on the function of myofibroblasts derived from an infarcted area. The myocardial infarction model was created by ligating the left anterior descending artery in rats. Two weeks after the operation, α-smooth muscle actin (α-SMA)-positive myofibroblasts were isolated from the infarcted area. Endostatin significantly increased the proliferation and migration of myofibroblasts in vitro. On the other hand, endostatin had no effect on the production of type I collagen, a major ECM protein produced by myofibroblasts. Endostatin activated Akt and extracellular signal-regulated kinase (ERK), and the pharmacological inhibition of these signaling pathways suppressed the endostatin-induced proliferation and migration. A knockdown of the *COL18A1* gene in the myocardial infarction model rats using small interference RNA (siRNA) worsened the cardiac function concomitant with wall thinning and decreased the α-SMA-positive myofibroblasts and scar formation compared with that of control siRNA-injected rats. In summary, we demonstrated for the first time that endostatin might be an important factor in the healing process after myocardial infarction through the activation of myofibroblasts.

## 1. Introduction

Functional disorder of organs induces tissue fibrosis which is characterized by an accumulation of extracellular matrix (ECM), including collagen, elastin, and fibronectin [1,2,3]. Moderate fibrosis is required for the healing process to maintain the structure and function of the damaged organs [1,4]. During the development of myocardial fibrosis, cardiac fibroblasts differentiate into their contractile phenotype, myofibroblasts, which express α-smooth muscle actin (α-SMA). Myofibroblasts are involved in the fibrosis through the production of ECM proteins (e.g., type I and type III collagen), ECM degrading enzymes (e.g., matrix metalloproteinases (MMPs)), pro-inflammatory cytokines (e.g., interleukin (IL)-1,6) and growth factors (e.g., epidermal growth factor and hepatocyte growth factor) [5,6,7,8,9,10]. Excessive proliferation and ECM production of myofibroblasts by the pathological activation of fibroblasts leads to the deterioration in myocardial compliance, dystelectasis, and cardioplegia [5]. After myocardial infarction, the moderate activation of myofibroblasts is necessary in the wound healing process through proliferation, migration, and ECM production. Therefore, the inadequate activation of myofibroblasts causes a poor scar formation and leads to cardiac dilatation, contractile dysfunction, and rupture [5]. These observations suggest that the regulation of myofibroblasts function might be an attractive therapeutic target for myocardial infarction.

Type XVIII collagen, a member of the multiplexin family, is mainly localized in the vascular basement membrane, which is cleaved by various proteases including MMPs, cathepsins, and elastase [11,12,13,14,15]. Endostatin, a 20 kDa cleaved C-terminal fragment of type XVIII collagen, was first separated from the culture medium of a murine hemangioendothelioma cell line [14]. Endostatin exerts an anti-angiogenic effect through the inhibition of migration and proliferation and the induction of apoptosis in vascular endothelial cells [14,16,17]. Subsequently, it inhibits the proliferation of tumor cells by suppressing the supply of nutrition and oxygen from blood vessels [18]. From the anti-tumor effect, zinc binding recombinant endostatin in the N-terminus (ZBP-endostatin; its trade name is Endostar) is approved by the State Food and Drug Administration of China for the treatment of non-small-cell lung cancer [19]. Endostatin also has pleiotropic effects including anti-hypertensive, anti-fibrotic, and anti-inflammatory effects [20,21,22].

The serum level of endostatin is increased in the patients with cardiovascular diseases [23,24]. Expression level of endostatin in cardiac tissue has been reported to be increased in the experimental cardiac disease models, such as pressure overload-induced cardiac hypertrophy [25,26] and myocardial infarction [27,28]. An anti-endostatin antibody-treatment has been reported to exacerbate the cardiac remodeling of non-infarcted area after myocardial infarction in rats [27]. We have recently reported that endostatin promotes the proliferation, migration, and wound-healing functions of cardiac fibroblasts derived from normal rats [29]. However, the pathological role of endostatin in the healing process of the infarcted area has not been clarified. In the present study, we investigated whether endostatin is responsible for the remodeling of the infarcted area after myocardial infarction through the modulation of biological functions of myofibroblasts.

## 2. Results

### 2.1. Effect of Endostatin on Proliferation of Myofibroblasts

**Figure 1:**
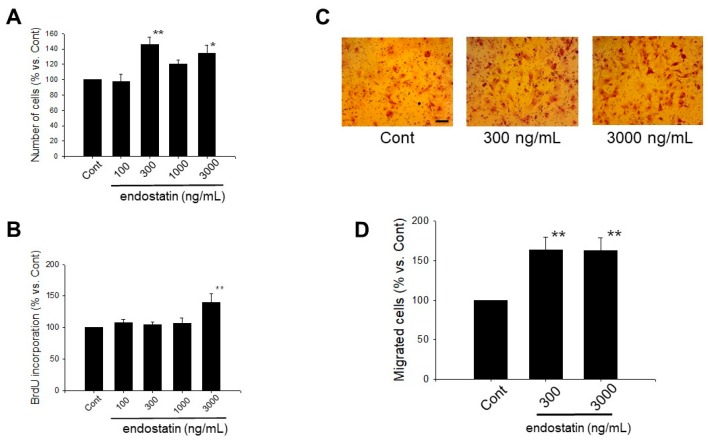
Endostatin induced proliferation and migration of myofibroblasts. (**A**) After myofibroblasts were grown to 30–50% confluence, they were stimulated with endostatin (100–3000 ng/mL) for 48 h. Control (Cont) was treated with a vehicle of endostatin (citrate-phosphate buffer). The number of viable cells was counted by a colorimetric method using a cell counting kit-8, and the normalized cell number relative to Cont are shown as mean ± standard error of the mean (S.E.M.) (*n* = 5). *, *p* < 0.05, **, *p* < 0.01 vs. Cont; (**B**) After the cells were seeded at a density of 1 × 10^4^ cells/well in a 96-well culture plate and starved (in 0.5% fetal bovine serum (FBS)), they were treated with endostatin (100–3000 ng/mL) or vehicle for 24 h. The cells were incubated with a bromodeoxyuridine (BrdU) reagent (20 µL/well) for 24 h, and BrdU incorporation was detected by a colorimetric method using an anti-BrdU antibody and tetramethylbenzidine solution. The normalized BrdU incorporation relative to Cont are shown as mean ± S.E.M. (*n* = 8). **, *p* < 0.01 vs. Cont; (**C**,**D**) Migration of myofibroblasts was determined by a Boyden chamber assay. The cells at a density of 1 × 10^5^ cells/100 µL serum free medium were seeded in the upper-chamber coated with 2% gelatin, and endostatin (300, 3000 ng/mL) or vehicle was treated in the bottom well for 24 h; (**C**) Representative pictures of Giemsa-stained migrated cells are shown. Scale bar: 100 µm. (**D**) The normalized migrated cell number relative to Cont are shown as mean ± S.E.M. (*n* = 7). **, *p* < 0.01 vs. Cont.

We first investigated the effect of endostatin (100–3000 ng/mL, 48 h) on cell morphology. Endostatin had no cytotoxicity on myofibroblasts (*n* = 5). Next, we examined the effect of endostatin on proliferation of myofibroblasts. Endostatin (48 h stimulation) significantly increased the number of cells, and the maximal effect was observed at 300 ng/mL (endostatin 300 ng/mL: *p* < 0.01 vs. control (Cont); endostatin 3000 ng/mL: *p* < 0.05 vs. Cont, *n* = 5) (Figure 1A). Additionally, we examined the effect of endostatin (100–3000 ng/mL, 24 h) on bromodeoxyuridine (BrdU) incorporation of myofibroblasts. Endostatin significantly increased BrdU incorporation (DNA synthesis activity) at 3000 ng/mL (*p* < 0.01 vs. Cont, *n* = 8) (Figure 1B).

### 2.2. Effect of Endostatin on Migration of Myofibroblasts

We next examined the effect of endostatin on migration of myofibroblasts by a Boyden chamber assay. Endostatin (300, 3000 ng/mL, 24 h stimulation) significantly increased cell migration (*p* < 0.01 vs. Cont, *n* = 7) (Figure 1C,D).

### 2.3. Effect of Endostatin on ECM Production and MMPs Secretion of Myofibroblasts

**Figure 2:**
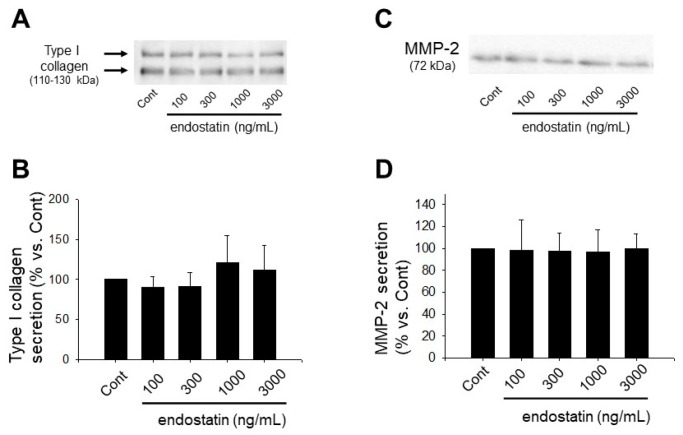
Endostatin had no effect on secretion of type I collagen and matrix metalloproteinase (MMP)-2 in myofibroblasts. Myofibroblasts were stimulated with endostatin (100–3000 ng/mL) or vehicle for 48 h, and culture medium was collected. Secretion of type I collagen and MMP-2 was determined by Western blotting. (**A**) Representative blot of type I collagen is shown. (**B**) Levels of secreted type I collagen were corrected by total cell protein concentration, and the normalized expression relative to Cont are shown as mean ± S.E.M. (*n* = 6); (**C**) Representative blot of MMP-2 is shown. (**D**) Levels of secreted MMP-2 were corrected by total cell protein concentration, and the normalized expression relative to Cont are shown as mean ± S.E.M. (*n* = 5).

We next examined secretion of type I collagen, a major ECM component produced by myofibroblasts, and MMP-2, an ECM-degrading enzyme, by Western blotting. Endostatin (100–3000 ng/mL, 48 h stimulation) had no effect on secretion of type I collagen (*n* = 6) (Figure 2A,B). Endostatin (100–3000 ng/mL, 48 h stimulation) also had no effect on MMP-2 secretion (*n* = 5) (Figure 2C,D).

### 2.4. Effect of Endostatin on Activation of Akt and Extracellular Signal-Regulated Kinase (ERK) in Myofibroblasts

**Figure 3:**
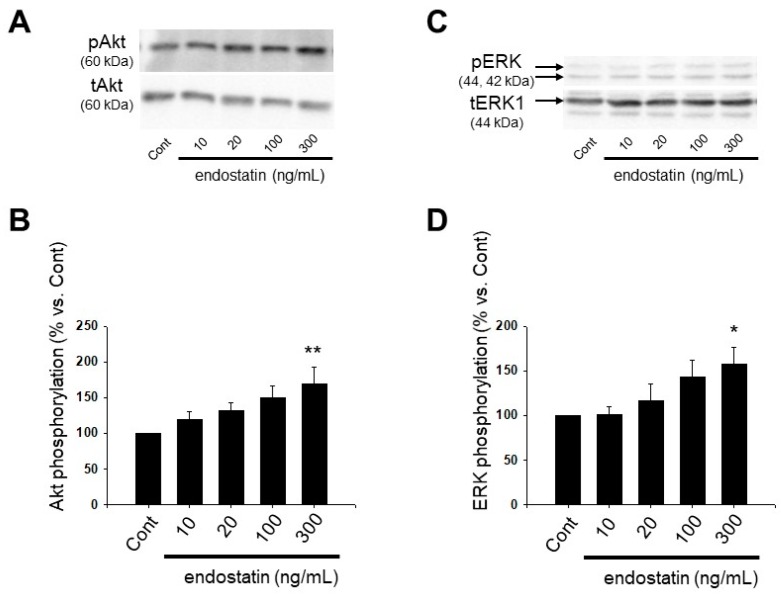
Endostatin induced phosphorylation of Akt and extracellular signal-regulated kinase (ERK) in myofibroblasts. Myofibroblasts were stimulated with endostatin (10–300 ng/mL, 60 min) or vehicle, and cell lysate was collected. Phosphorylation of Akt (at Ser473: **A**,**B**) and ERK (**C**,**D**) was detected by Western blotting. (**A**,**C**) Representative blots of phosphorylated proteins (pAkt: **A** pERK: **C**) and total proteins (tAkt: **A**; tERK1: **C**) for Akt and ERK are shown; (**B**,**D**) Levels of phosphorylated proteins were corrected by total proteins, and the normalized expression relative to Cont are shown as mean ± S.E.M. (Akt: *n* = 12, ERK: *n* = 11). *, *p* < 0.05, **, *p* < 0.01 vs. Cont.

We examined the effect of endostatin on phosphorylation of Akt and ERK by Western blotting. From the results of the cell counting assay and Boyden chamber assay (Figure 1A,C), 300 ng/mL endostatin seems to be a maximally effective dose for proliferation and migration of myofibroblasts. Thus, we examined the effect of 300 ng/mL endostatin. In a preliminary experiment, we observed that 300 ng/mL endostatin (10–360 min) increased phosphorylation of Akt and ERK maximally at 60 min (Akt: *n* = 7; ERK: *n* = 9). Then, we examined the dose-response of endostatin at 60 min. Endostatin (10–300 ng/mL, 60 min) increased phosphorylation of Akt (endostatin 300 ng/mL: *p* < 0.01 vs. Cont, *n* = 12) and ERK (endostatin 300 ng/mL: *p* < 0.05 vs. Cont, *n* = 11) in a concentration-dependent manner (Figure 3).

### 2.5. Endostatin Induced Proliferation and Migration of Myofibroblasts through Activation of Phosphoinositide 3-Kinase (PI3K)/Akt and Mitogen-Activated Protein Kinase (MAPK)/ERK Kinase (MEK)/ERK Pathway

**Figure 4:**
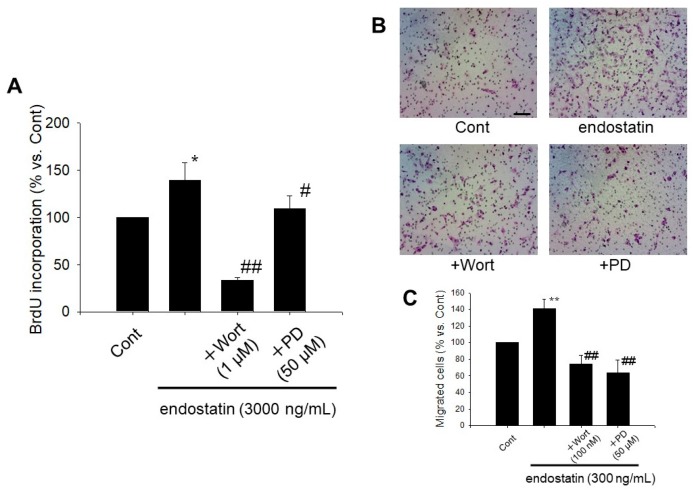
Wortmannin (a phosphoinositide 3-kinase inhibitor) or PD98059 (a mitogen-activated protein kinase/ERK kinase inhibitor) inhibited endostatin-induced proliferation and migration of myofibroblasts. (**A**) After the cells were seeded at a density of 1 × 10^4^ cells/well in a 96-well culture plate and starved (in 0.5% FBS), they were treated with endostatin (3000 ng/mL) or vehicle for 24 h in the presence or absence of wortmannin (Wort: 1 μM, 30 min pre-treatment) or PD98059 (PD: 50 μM, 30 min pre-treatment). The cells were incubated with a BrdU reagent (20 µL/well) for 24 h, and BrdU incorporation was detected by a colorimetric method using an anti-BrdU antibody and tetramethylbenzidine solution. The normalized BrdU incorporation relative to Cont are shown as mean ± S.E.M. (*n* = 9). *, *p* < 0.05 vs. Cont, #, *p* < 0.05, ##, *p* < 0.01 vs. endostatin-alone treatment; (**B**,**C**) Migration of myofibroblasts was determined by a Boyden chamber assay. The cells at a density of 1 × 10^5^ cells/100 µL serum free medium were seeded in the upper-chamber coated with 2% gelatin, and endostatin (300 ng/mL) or vehicle was treated in the bottom well for 24 h. Wortmannin (Wort: 100 nM) or PD98059 (PD: 50 µM) was pre-treated in both upper and lower chambers 30 min before endostatin-stimulation. (**B**) Representative pictures of Giemsa-stained migrated cells are shown. Scale bar: 100 µm. (**C**) The normalized migrated cell number relative to Cont are shown as mean ± S.E.M. (*n* = 6). **, *p* < 0.01 vs. Cont, ##, *p* < 0.01 vs. endostatin-alone treatment.

We next investigated whether endostatin induced proliferation and migration of myofibroblasts through the activation of Akt and ERK pathways by using wortmannin, a PI3K/Akt inhibitor, and PD98059, a MEK/ERK inhibitor [29,30,31]. Wortmannin (1 µM) or PD98059 (50 µM) significantly inhibited the endostatin (3000 ng/mL, 24 h)-induced BrdU incorporation (endostatin: *p* < 0.05 vs. Cont; +Wortmannin: *p* < 0.01 vs. endostatin; +PD98059: *p* < 0.05 vs. endostatin, *n* = 9) (Figure 4A). Wortmannin (100 nM) or PD98059 (50 µM) significantly inhibited the endostatin (300 ng/mL, 24 h)-induced migration (endostatin: *p* < 0.01 vs. Cont; +Wortmannin: *p* < 0.01 vs. endostatin; +PD98059: *p* < 0.01 vs. endostatin, *n* = 6) (Figure 4B,C).

### 2.6. A Knockdown of the COL18A1 Gene in the Infarcted Area by COL18A1 Small Interference (si) RNA Worsened Cardiac Function and Decreased α-SMA-Positive Myofibroblasts

We used siRNA against the *COL18A1* gene, the effectiveness of which on endostatin expression was previously confirmed in hearts of rats [32]. *COL18A1* siRNA had no significant effect on the body weight of rats (Table 1).

**Figure 5:**
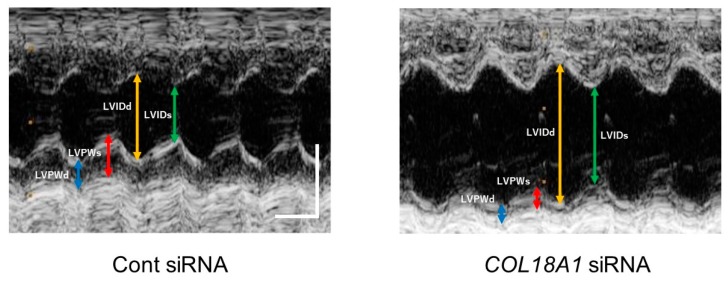
The M-mode echocardiographic image from parasternal short axis view of the left ventricle at the level of papillary muscles in siRNA-injected myocardial infarction model rats. After the ligation of the left anterior descending artery (LAD) in rats, sixteen μg of *COL18A1* siRNA or Cont siRNA was injected into the infarcted myocardium. Two weeks after the operation, echocardiography was performed. Representative images of M-mode on the parasternal short axis view of the left ventricle are shown (Cont siRNA: *n* = 6, *COL18A1* siRNA: *n* = 6). Scale bar: 100 msec (horizontal) and 5 mm (vertical). LVIDd: left ventricular internal dimension at end-diastole, LVIDs: Left ventricular internal dimension at end-systole, LVPWd: Left ventricular posterior wall at end-diastole, LVPWs: Left ventricular posterior wall at end-systole.

In the echocardiographic analysis, left ventricular (LV) mass was not changed by a *COL18A1* siRNA injection. On the other hand, LV internal dimension at end-systole (LVIDs) (*n* = 6, *p* < 0.01), interventricular septum (IVS)/LV posterior wall (LVPW) (*n* = 6, *p* < 0.05) and end-systolic volume (ESV) (*n* = 6, *p* < 0.05) were increased by a *COL18A1* siRNA injection. In addition, LVPW at end-diastole (LVPWd) (*n* = 6, *p* < 0.01), end-systole (LVPWs) (*n* = 6, *p* < 0.01) and percentage LVPW thickness (LVPW%) (*n* = 6, *p* < 0.05) were decreased by a *COL18A1* siRNA injection. These observations demonstrated that the endostatin knockdown induced thinning and dilatation of LV. The decrease of fractional shortening (FS) (*n* = 6, *p* < 0.01) and ejection fraction (EF) (*n* = 6, *p* < 0.01) indicated that the endostatin knockdown worsened the cardiac function (Figure 5 and Table 2). 

**Figure 6:**
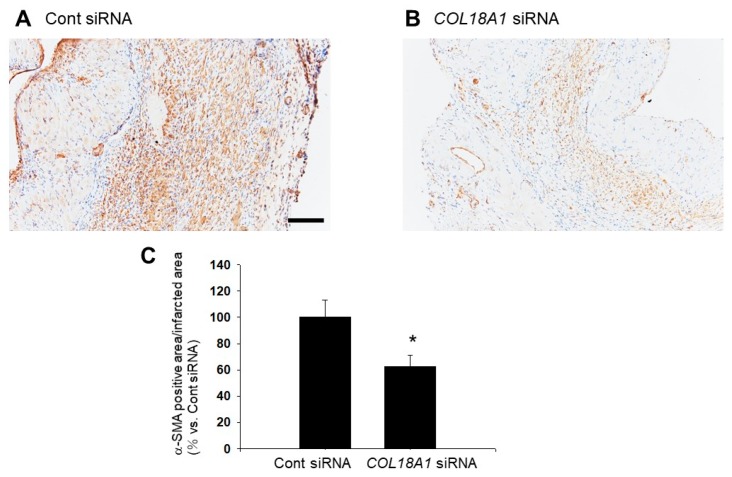
Distribution of α-smooth muscle actin (α-SMA)-positive myofibroblasts in infarcted area of siRNA-injected myocardial infarction model rats. After ligation of the left anterior descending artery (LAD) in rats, sixteen µg of *COL18A1* siRNA or Cont siRNA was injected into the infarcted myocardium. Two weeks after the operation, the heart was isolated and then thin paraffin sections (4 µm) were made. (**A**,**B**) Representative pictures of the myocardium stained with a specific antibody against α-SMA are shown (**A**: Cont siRNA; **B**: *COL18A1* siRNA). Nuclei were counterstained with hematoxylin (Cont siRNA: *n* = 4; *COL18A1* siRNA: *n* = 6). Scale bar: 200 µm; (**C**) The α-SMA positive area was calculated, and the normalized area relative to Cont siRNA are shown as mean ± S.E.M. (Cont siRNA: *n* = 4, *COL18A1* siRNA: *n* = 6). *, *p* < 0.05 vs. Cont siRNA.

We next investigated the distribution of α-SMA-positive myofibroblasts [33] in the infarcted area after myocardial infarction. The α-SMA-positive myofibroblasts were observed throughout the infarcted area in Cont siRNA-injected rats (Figure 6A). On the other hand, the α-SMA-positive myofibroblasts were sparsely expressed in the infarcted area in *COL18A1* siRNA-injected rats (Figure 6B). The α-SMA-positive area was significantly decreased by a *COL18A1* siRNA injection (Cont siRNA: *n* = 4; *COL18A1* siRNA: *n* = 6, *p* < 0.05) (Figure 6C).

**Figure 7:**
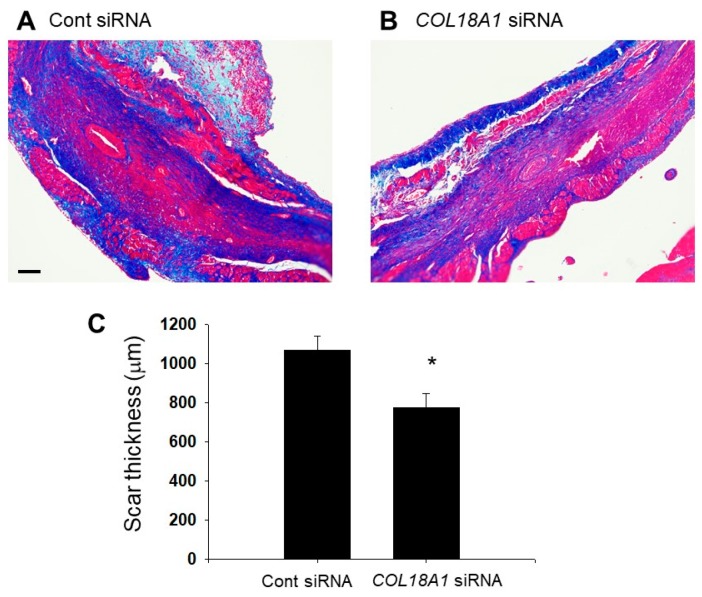
Azan staining for scar formation in infarcted area of siRNA-injected myocardial infarction model rats. After ligation of the LAD in rats, sixteen µg of *COL18A1* siRNA or Cont siRNA was injected into the infarcted myocardium. Two weeks after the operation, the heart was isolated and then thin paraffin sections (4 µm) were made. (**A**,**B**) Representative Azan stained pictures of the infarcted area are shown (**A**: Cont siRNA; **B**: *COL18A1* siRNA). Blue: collagen fibers; Red: muscle fibers. Scale bar: 100 µm; (**C**) Scar thickness was measured and are shown as mean ± S.E.M. (Cont siRNA: *n* = 4, *COL18A1* siRNA: *n* = 5). * *p* < 0.05 vs. Cont siRNA.

We finally examined the effect of endostatin on scar formation by Azan staining. The thickness of the scar tissue composed of collagen fibers (blue) in the infarcted area was significantly decreased by a *COL18A1* siRNA injection compared with that of Cont siRNA siRNA-injection (Cont siRNA: *n* = 4; *COL18A1* siRNA: *n* = 5, *p* < 0.05) (Figure 7).

## 3. Discussion

In this study, we revealed for the first time that endostatin induced proliferation and migration of myofibroblasts derived from the infarcted areas of myocardial infarction model rats via the activation of Akt and ERK. A knockdown of endostatin worsened the cardiac function concomitant with wall thinning and decreased α-SMA positive myofibroblasts in the infarcted area. It is thus suggested that endostatin might have an important role in the scar formation and contractile stability after myocardial infarction through the activation of myofibroblasts.

In this study, we used 100–3000 ng/mL recombinant mouse endostatin. The blood concentration of endostatin in healthy individuals is ~88.1 ng/mL [34]. In contrast, the serum concentration of endostatin in patients with chronic heart failure is ~229 ng/mL [24]. The concentration of endostatin in coronary sinus serum is significantly elevated in patients with coronary heart disease compared with normal subjects (median 79.7 ng/mL vs. median 49.6 ng/mL) [23]. The serum endostatin level has been reported to be significantly increased at 24 h after myocardial infarction (>100 ng/mL) compared with sham operated rats (<50 ng/mL). In addition, the intensity of immunofluorescent staining for endostatin/collagen XVIII was markedly upregulated in cardiomyocytes in the infarcted area [27]. The myofibroblasts used in this study were isolated from the infarcted area. Since the endostatin level in the infarcted area might be higher than the serum level, the endostatin concentrations (100–3000 ng/mL) used in the present study would be within the pathological ranges. 

In this study, endostatin induced proliferation and migration of myofibroblasts (Figure 3). Endostatin has anti-angiogenic effect through the inhibition of proliferation and migration of vascular endothelial cells via inhibiting phosphorylation of focal adhesion kinase through binding integrin α_5_β_1_ or the antagonism of vascular endothelial growth factor receptor [35,36] Alahuhta et al. reported that endostatin induced proliferation of HSC-3 carcinoma cells, a human tongue squamous cell carcinoma cell line [37]. Huang et al. reported that 50 μg/mL recombinant human endostatin inhibited proliferation of synovial fibroblasts in rats with adjuvant arthritis but not normal rats [38]. On the other hand, we previously reported that endostatin induced proliferation and migration of cardiac fibroblasts [29]. Therefore, endostatin might exert different effects on proliferation and migration depending on the cell types. In the present study, endostatin promoted phosphorylation of Akt (Ser473) and ERK (Figure 3), and inhibition of PI3K/Akt and MEK/ERK pathways by using wortmannin and PD98059 inhibited the endostatin-induced proliferation and migration, respectively (Figure 4). It is known that activation of Akt and ERK is involved in proliferation and migration of myofibroblasts [39,40,41,42]. Thus, it is suggested that endostatin induced proliferation and migration via activation of Akt and ERK in cardiac myofibroblasts. Wenzel et al. reported that endostatin accelerated relaxation response via production of nitric monoxide and stimulation of the PI3K/Akt pathway in isolated aortic tissue [43]. Endostatin stimulates reactive oxygen species (ROS) through the increase of intracellular ceramide concentration in bovine coronary artery-derived endothelial cells [44]. Yang et al. reported that plumbagin activated ERK1/2 and Akt via the production of ROS in 3T3-L1 cells, a mouse embryonic fibroblast cell line [45] and that *N*-acetylcysteine (NAC), an antioxidant, inhibited the plumbagin-induced Akt phosphorylation [45]. Moreover, we previously revealed that NAC inhibited endostatin-induced Akt activation in rat cardiac fibroblasts [29]. Thus, it is supposed that endostatin activates PI3K/Akt and MEK/ERK pathways through the ROS production in myofibroblasts, similar to the case in cardiac fibroblasts.

It has been reported that endostatin expression increased in the infarcted area of rat myocardial infarction model [27,28]. Therefore, we examined the effect of endostatin knockdown by *COL18A1* siRNA on the remodeling of infarcted area. Interestingly, a knockdown of *COL18A1* gene caused a remarkable thinning of the infarcted area, which was characterized by the decrease of LVPWd, LVPWs, and LVPW% and the increase of LVIDs, IVS/LVPW, and ESV (Figure 5 and Table 2). The endostatin knockdown also worsened cardiac function, which was characterized by the decrease of FS and EF (Figure 5 and Table 2). In addition, the α-SMA-positive myofibroblasts in the infarcted area of the *COL18A1* siRNA-injected rats appeared sparse compared with control siRNA-injected rats (Figure 6). The insufficient proliferation and migration of myofibroblasts after myocardial infarction is thought to induce thinning of the wall, cardiac dilatation, cardiac dysfunction, and ventricular wall rupture [46]. Furthermore, scar thickness in the infarcted area of *COL18A1* siRNA-injected rats were decreased compared with control siRNA-injected rats (Figure 7). Therefore, it is suggested that endostatin is necessary for the sufficient proliferation and migration of myofibroblasts in the infarcted area, which contributes to the scar formation. The limitation of this study is that we used a *COL18A1* siRNA to knockdown endostatin. Further study is needed to investigate the influence of *COL18A1*-specific gene knockdown on the healing process after myocardial infarction.

In conclusion, we revealed for the first time that endostatin might regulate the healing process in an infarcted area after myocardial infarction through the activation of proliferation and migration of myofibroblasts. These findings indicate endostatin as a new therapeutic target for myocardial infarction.

## 4. Materials and Methods

### 4.1. Reagents and Antibodies

Reagent sources were as follows: Recombinant mouse endostatin (Sigma-Aldrich, St. Louis, MO, USA), PD98059 (Cayman, Ann Arbor, MI, USA), wortmannin (Wako, Osaka, Japan).

Antibodies sources were as follows: anti-phospho-Akt (Ser473), anti-total Akt, anti-phospho-ERK (Cell Signaling Technology, Beverly, MA, USA), anti-total-ERK1, anti-endostatin (Santa Cruz Biotech, Santa Cruz, CA, USA), anti-type I collagen (Rockland immunochemicals, Gilbertsville, PA, USA), anti-MMP-2 (Kyowa pharma chemical, Toyama, Japan), anti-total-glyceraldehyde 3-phosphate dehydrogenase (GAPDH) (Wako), α-SMA (Dako, Glostrup, Denmark), anti-rabbit immunoglobulin G (IgG) horseradish peroxidase linked whole antibody, anti-mouse IgG horseradish peroxidase linked whole antibody (Amersham Bioscences, Buckinghamshire, UK).

### 4.2. Myocardial Infarction Model

All animal studies were approved by the President of Kitasato University through the judgment by the Institutional Animal Care and Use Committee of Kitasato University (Approval No. 14-126 (13 March 2015), 15-020 (4 March 2016), 16-031 (5 December 2016) and 17-081 (21 April 2017)). Adult male Wistar rats (7–10-week old; CLEA Japan, Tokyo, Japan) were cared for in accordance with the National Institutes of Health Guide for the Care of Laboratory Animals and the Guideline for Animal Care and Treatment of the Kitasato University. Coronary artery ligation of the rats was performed to create a myocardial infarction model as described previously [42]. After the rats were anesthetized with isoflurane by using a vaporizer, endotracheal intubation was connected to a ventilator with a respiratory rate of 100 times per min and tidal volume of 5 mL. Buprenorphine (0.005 mg/100 g) was subcutaneously administered for a preoperative analgesia. Then, a left thoracotomy between third or fourth intercostal space was performed to expose the heart, and the proximal left anterior descending artery was permanently ligated using a 6-0 nylon suture. After the chest was closed, rats recovered. 

We also performed a knockdown of endostatin in the infarcted area by using an siRNA as described previously [32,47]. It was previously confirmed that the *COL18A1* siRNA used in this study decreased endostatin expression in hearts of rats [32]. After the coronary ligation, sixteen μg of *COL18A1* siRNA or control siRNA was injected into the infarcted myocardium. After the chest was closed, rats recovered and were cared for 14 days. The sequence of *COL18A1* siRNA was as follows: sense 5′-UCGUCAACCUGAAGGAUGAdTdT-3′ and antisense 5′-UCAUCCUUCAGGUUGACG AdTdT-3′.

### 4.3. Isolation of Myofibroblasts from the Areas of Myocardial Infarction

Myofibroblasts were isolated from the areas of myocardial infarction as described previously [42]. The myocardial infarction model rats were cared for 2 weeks and the hearts were isolated under pentobarbital (80 mg/kg, intraperitoneally injection) anesthesia. The isolated heart was washed with oxygenated Krebs-Henseleit solution (119 mM NaCl, 4.8 mM KCl, 2.5 mM CaCl_2_, 1.2 mM KH_2_PO_4_, 1.2 mM MgSO_4_, 24.9 mM NaHCO_3_, 10.0 mM Glucose) for an exsanguination, and the infarcted area was isolated. The infarcted tissue was cut into 4–5 mm pieces for isolation of myofibroblasts and washed with Tris buffered saline (TBS, pH 7.4) and Dulbecco’s Modified Eagle’s Medium (DMEM, Sigma Aldrich). Then, the piece of tissue was put on a 100 mm-culture dish and incubated in DMEM containing 10% fetal bovine serum (FBS: HyClone/GE Healthcare, Little Chalfont, UK) at 37 °C in 5% CO_2_. The medium was changed every 2–3 days. The piece of tissue was removed 10 days after culture, and the migrated and proliferated cells around the tissue were trypsinized and passaged. By using an immunofluorescence staining, we confirmed that the isolated cells were stained positively with antibody against α-SMA, vimentin, and collagen type I but not CD31, which was consistent with the myofibroblasts phenotype as described previously [42]. Passage 2–9 cells were used and a 24 h starvation in DMEM containing 0.5% FBS was performed before the stimulation.

### 4.4. Cell Proliferation Assay

Cell proliferation was measured by using Cell counting kit-8 (CC8, Dojindo, Kumamoto, Japan) as described previously [42]. After the cells were grown to 30–50% confluence in a 6-well culture plate, they were stimulated with endostatin (100–3000 ng/mL) for 48 h. After washing with TBS, the cells were treated with the CC8 solution (25 μL/0.5 mL medium) for 1 h at 37 °C. The absorbance of media at 485 nm was measured using a standard microplate reader (Tristar, Berthold Technologies, Bad Wildbad, Germany).

BrdU incorporation assay was also performed to detect the cell proliferation by using a BrdU cell proliferation assay kit (Exalpha biologicals Inc., Shirley, MA, USA) as described previously [48]. After the cells were seeded at a density of 1 × 10^4^ cells/well in a 96-well culture plate and incubated for 24 h at 37 °C and 5% CO_2_, they were starved with DMEM containing 0.5% FBS for 24 h. After the cells were stimulated with endostatin (100–3000 ng/mL) for 24 h in the presence or absence of wortmannin (1 μM) or PD98059 (50 μM), they were incubated with a BrdU reagent (20 µL/well) for 24 h. After the cells were fixed with a fixing solution (50 µL/well) for 30 min, they were incubated with an anti-BrdU antibody for 1 h and subsequently incubated with a horseradish peroxidase conjugated anti-mouse IgG (1:2000 dilution) for 30 min. After the cells were treated with tetramethylbenzidine solution (50 µL/well) for 30 min, the reaction was stopped by adding a stop solution. The absorbance at 450 nm was measured by using a microplate reader (Berthold Technologies).

### 4.5. Boyden Chamber Assay

Cell migration was detected by a Boyden chamber assay with Transwell chambers (pore size: 8.0 µm, Corning Incorporated, Arizona, AZ, USA) coated with 2% gelatin as described previously [42]. The cells at density of 1 × 10^5^ cells/100 μL were plated in the upper-chamber, and endostatin (300, 3000 ng/mL) was treated in the bottom well. Then they were incubated for 24 h in the presence or absence of wortmannin (100 nM) or PD98059 (50 μM) in both the upper and bottom wells. The migrated cell number in three randomly selected areas was counted with a phase-contrast microscope (CKX-41, OLYMPUS, Tokyo, Japan) equipped with a universal serial bus (USB) camera (MIR-MDCE-5C, Bio Medical Science, Tokyo, Japan), and the normalized cell number relative to vehicle-treated control was evaluated.

### 4.6. Western Blotting

Western blotting was performed to detect the expression and phosphorylation of proteins as described previously [42]. After the stimulation with endostatin (100–3000 ng/mL, 10 min–48 h), the culture media was collected and the total cell lysate was extracted by a cell lysis buffer (Cell Signaling Technology) with protease inhibitors (Nakalai Tesque, Kyoto, Japan). Ten μg of total cell lysate or 20 µL culture media was separated by sodium dodecyl sulfate-polyacrylamide gel electrophoresis (7.5–14%) (80–120 V, 1.5–2 h) and transferred to a nitrocellulose membrane (Pall Corporation, Ann Arbor, MI, USA) (400 mA, 1.5 h). After the membranes were blocked with 0.5% skim milk (for total proteins) or 3% bovine serum albumin (for phosphorylated proteins), the membranes were incubated with a primary antibody at 4 °C overnight and then reacted with horseradish peroxidase-conjugated secondary antibody (1:10,000 dilution in TBS, 1 h). The chemiluminescence signal was detected by the EZ-ECL system (Biological Industries, Kibbutz Beit Haemek, Israel). Equal loading of protein was confirmed by measuring total-protein or total-actin. The detected bands were analyzed using CS analyzer 3.0 software (AE-6972; ATTO, Tokyo, Japan).

### 4.7. Echocardiography

Echocardiography was performed under isoflurane anesthesia with iE33 (Philips, WA, USA) as described previously [27,32]. The heart rate of rats was constantly regulated by anesthetic depth, and the M-mode image was obtained from the parasternal short axis view. IVS, LVID, LVPW, EDV, ESV, LV mass, FS, EF, and heart rate were measured in both diastolic and systolic phases from the M-mode images.

### 4.8. Immunohistochemical Staining

After the isolated heart was fixed in 10% neutral buffered formalin, thin paraffin sections (4 µm) were immunohistochemically stained as described previously [42]. First, the deparaffinized section was heated by using a microwave for antigen activation and incubated in methanol with 0.3% H_2_O_2_ for 20 min to block endogenous peroxidase activity. Next, the section was blocked with 5% normal goat serum and incubated with primary antibody against α-SMA (1:100 dilution) at 4 °C overnight. After washing with TBS, the section was incubated in biotinylated link (Dako) for 60 min and next in streptavidin-horseradish peroxidase (Dako) for 30 min at room temperature. Then, expression of α-SMA was visualized by a liquid DAB+substrate chromogen system (Dako). The images were obtained using a light microscope (BX-51, OLYMPUS) equipped with a microscope digital camera (DP74, OLYMPUS). The α-SMA-positive area to infarcted area was calculated by ImageJ software.

### 4.9. Azan Staining

After the isolated heart was fixed in 10% neutral buffered formalin, thin paraffin sections (4 µm) were applied with Azan staining as described previously [49]. The deparaffinized section was soaked in 5% potassium dichromate solution for 60 min and stained with Azocarmine G (Waldeck, Division Chroma, Münster, Germany) over night. Next, the section was soaked in 3% 12-tungsto-(IV)-phosphoric acid *n*-hydrate solution for 15 min and stained with aniline blue and orange G solution (Waldeck, Division Chroma) for 60 min. The images were obtained using a light microscope (BX-51) equipped with a microscope digital camera (DP74). The scar thickness was measured in the point of the thickest scar tissue by cellSens Imaging Software (OLYMPUS).

### 4.10. Statistical Analysis

Data are presented as means ± the standard error of the mean. In two-group comparison, statistical analyses were performed by using Student’s *t* test. In multi-group comparison, statistical analyses were performed by one-way ANOVA followed by Dunnet’s post hoc test (single treatment of endostatin) and Bonferroni’s post hoc test (co-treatment of endostatin and inhibitors). A value of *p* < 0.05 was considered statistically significant.

## Figures and Tables

**Table 1:** Body weight (BW) in small interference (si) RNA-injected myocardial infarction model rats.

Parameters	Cont siRNA (*n* = 6)	*COL18A1* siRNA (*n* = 6)
BW (before operation)	265.8 ± 16.4 g	270.9 ± 17.2 g
BW (2 weeks after operation)	318.6 ± 10.0 g	310.7 ± 13.2 g
ΔBW	52.8 ± 6.9 g	39.8 ± 5.9 g

BW of Cont siRNA- or *COL18A1* siRNA-injected myocardial infarction model rats. The data are shown as mean ± standard error of the mean (S.E.M.). ΔBW: BW (2 weeks after operation)—BW (before operation).

**Table 2:** Echocardiographic parameters in siRNA-injected myocardial infarction model rats.

Parameters	Unit	Cont siRNA(*n* = 6)	*COL18A1* siRNA (*n* = 6)
IVSd	cm	0.18 ± 0.01	0.17 ± 0.01
LVIDd	cm	0.72 ± 0.04	0.85 ± 0.04
LVPWd	cm	0.20 ± 0.01	0.15 ± 0.01 **
IVSs	cm	0.30 ± 0.01	0.29 ± 0.02
LVIDs	cm	0.40 ± 0.02	0.57 ± 0.04 **
LVPWs	cm	0.29 ± 0.02	0.19 ± 0.02 **
EDV (MM-Teich)	mL	0.89 ± 0.12	1.38 ± 0.21
IVS/LVPW (MM)		0.94 ± 0.07	1.17 ± 0.06 *
LV Mass (Cubed)	g	1.4 ± 0.1	1.4 ± 0.1
IVS% (MM)	%	69.0 ± 9.3	69.7 ± 7.6
ESV (MM-Teich)	mL	0.16 ± 0.02	0.49 ± 0.11 *
FS (MM-Teich)	%	44.5 ± 2.7	32.9 ± 2.0 **
EF (MM-Teich)	%	80.0 ± 2.9	66.5 ± 2.7 **
LVPW% (MM)	%	51.7 ± 5.8	26.3 ± 8.3 *
Heart rate	bpm	405.9 ± 9.7	376.2 ± 7.4 *

Two weeks after the operation of myocardial infarction, echocardiography was performed. IVSd: Interventricular septum at end-diastole, LVIDd: left ventricular internal dimension at end-diastole, LVPWd: Left ventricular posterior wall dimension at end-diastole, IVSs: Interventricular septum at end-systole, LVIDs: Left ventricular internal dimension at end-systole, LVPWs: Left ventricular posterior wall at end-systole, EDV: End-diastolic volume, IVS%: percentage IVS thickness, ESV: End-systolic volume, FS: Fractional shortening, EF: Ejection fraction, LVPW%: percentage LVPW thickness. The data are shown as mean ± S.E.M. *, *p* < 0.05, **, *p* < 0.01 vs. Cont siRNA.

## Abbreviations

ECM	extracellular matrix
α-SMA	α-smooth muscle actin
MMPs	matrix metalloproteinases
IL	interleukin
S.E.M.	standard error of the mean
ERK	extracellular signal-regulated kinase
PI3K	phosphoinositide 3-kinase
MAPK	mitogen-activated protein kinase
MEK	MAPK/ERK kinase
siRNA	small interference RNA
LAD	left anterior descending artery
GAPDH	glyceraldehyde 3-phosphate dehydrogenase
BW	body weight
LV	left ventricular
LVIDd	LV internal dimension at end-diastole
LVIDs	LV internal dimension at end-systole
IVSd	interventricular septum at end-diastole
IVSs	interventricular septum at end-systole
IVS%	percentage IVS thickness
LVPWd	LV posterior wall at end-diastole
LVPWs	LV posterior wall at end-systole
LVPW%	percentage LVPW thickness
EDV	end-diastolic volume
ESV	end-systolic volume
FS	fractional shortening
EF	ejection fraction
ROS	reactive oxygen species
NAC	*N*-acetylcysteine
TBS	tris buffered saline
DMEM	Dulbecco’s Modified Eagle’s Medium
FBS	fetal bovine serum
CC8	cell counting kit-8
